# Association between low fatty acid levels and platelet count in infants with Retinopathy of Prematurity

**DOI:** 10.1111/apa.15406

**Published:** 2020-06-29

**Authors:** Pia Lundgren, Anders K. Nilsson, Gunnel Hellgren, Aldina Pivodic, Lois E.H. Smith, Ann Hellström

**Affiliations:** 1 Section for Ophthalmology, Department of Clinical Neuroscience, Institute of Neuroscience and Physiology, Sahlgrenska Academy, University of Gothenburg, Gothenburg, Sweden; 2 School of Medical Sciences, Faculty of Medicine and Health, Örebro Univeristy, Örebro, Sweden; 3 Institute of Biomedicine, Sahlgrenska Academy, University of Gothenburg, Gothenburg, Sweden; 4 Statistiska konsultgruppen, Gothenburg, Sweden; 5 The Department of Ophthalmology, Boston Children’s Hospital, Harvard Medical School, Boston, MA, USA; 6 Region Västra Götaland, Sahlgrenska University Hospital, Department of Ophthalmology, Mölndal, Sweden

Numerous factors are involved in retinopathy of prematurity (ROP) and these differ between preterm infants and fetuses. Sight-threatening pathological retinal neovascularisation may occur during the second phase, due to increased expression of pro-angiogenic factors like vascular endothelial growth factor-A (VEGF-A).

Low platelet counts and thrombocytopenia are independent risk factors for ROP. Experimental mice studies have suggested that platelets release stimuli that cause downregulation of retinal VEGF-A and supress neovascularisation.^1^ Low levels of long-chain polyunsaturated fatty acids (LC-PUFAs), like docosahexaenoic acid (DHA) and arachidonic acid (AA), have been associated with ROP.^2^ LC-PUFAs have enhanced platelet formation from mature megakaryocytes in vitro.^3^ Low platelet counts and low levels of LC-PUFAs have been associated with ROP, but their inter-relationship has not been evaluated.

This was a prospective study of 78 infants born at Sahlgrenska University Hospital, Gothenburg, Sweden, before 28 weeks of gestation in 2013–2015. We assessed their longitudinal postnatal platelet counts and serum phospholipid-bound fractions of DHA and AA^4^ in relation to ROP. Blood samples for LC-PUFA analysis and platelet counts were taken from birth until 40 weeks of postmenstrual age (PMA). Thrombocytopenia was any platelet count <100 × 10^9^/L. LC-PUFA levels and platelet counts were used to calculate weekly Pearson correlations for PMAs from 28 to 40 weeks. Two-tailed tests were used and .05 was significant. The analyses were performed using SAS software, version 9.4 (SAS Institute Inc.).

Table 1 shows the infants’ characteristics and the correlations between DHA, AA and platelet counts. Of these, 28% developed severe ROP requiring treatment at a median PMA of 36.8 (interquartile range 35.4–38.9) weeks and exhibited thrombocytopenia more frequently (82% versus 36%, *P* < .001) than those with no, or less severe, ROP. Detailed data about their serum fractions of AA and DHA have previously been published.^4^ Briefly, DHA decreased by 1.14 (95% confidence interval 0.93–1.36, *P* < .0001) mol% and AA decreased by 7.5 (6.8–8.1, *P* < .0001) mol% during the first postnatal week. DHA then increased slowly and AA remained low. The associations between DHA, AA and the predicted platelet counts were *r* = .32 (range 0.23–0.43, *P* = .0008) and r = 0.23 (0.16–0.34, *P* = .0045) respectively. They were markedly higher in infants with severe ROP (Table 1), particularly for DHA between 32 and 36 weeks of PMA (range 0.59–0.66), when neovascularisation may occur (Table 1, Figure S1). AA presented an overall weaker association than DHA.

We believe that no associations between platelet counts and LC-PUFAs have previously been reported for extremely preterm infants. However, in the 1970s, Freidman et al reported thrombocytopenia in infants with essential fatty acid deficiency and long-time parenteral nutrition.^5^ We hypothesised that low LC-PUFA would interact negatively with platelet formation. Consequently, platelet derived anti-angiogenic stimuli were decreased which may have contributed to neovascularisation in the second ROP phase.

Retinal vascularisation involves a complex interaction and timing of pro-angiogenic or anti-angiogenic factors. In ROP, pathological neovascularisation occurs when retinal expression of VEGF-A is upregulated to meet the demands of the maturing neural retina. An ROP mouse model indicated that platelet alpha-granules released anti-angiogenic stimuli, causing downregulation of retinal VEGF-A transcripts and suppressed neovascularisation during the second ROP phase.^1^ Platelets are released from mature megakaryocytes in bone marrow through a series of events. Our positive association between LC-PUFAs and platelets was not surprising, as in vitro studies have shown that DHA and AA in culture media improved megakaryocyte migration, maintained platelet membrane structure and affected platelet survival.^3^

Postnatal LC-PUFA deficiency appears from week one of life and persists during the first few months in extremely preterm infants. Many studies have failed to show the benefits of LC-PUFA supplements in preterm infants. We emphasise the importance of analysing longitudinal serum fraction levels of LC-PUFA, to confirm the effect of supplementation before evaluating any benefits. As previously suggested, PMA-adjusted AA and DHA supplements could prevent morbidities. AA is the predominant LC-PUFA in the retina up to approximately 32 weeks of PMA, when retinal DHA increases and AA decreases. Retinal vascularisation and suppression of vacularisation was higher in phase two ROP in mice fed a DHA, rather than AA, enriched diet. We hypothesise that DHA supplements were especially important in later PMAs, due to a plausible synergistic pathogenesis between platelets and DHA in ROP development.

Further studies need to clarify the causality between DHA and AA fractions and platelet counts and address the timing, duration, dosage and optimal ratios of LC-PUFA supplements in extremely preterm infants. This could help to prevent morbidities like severe sight-threatening ROP.

## Supplementary Material

1

## Figures and Tables

**TABLE 1:** Clinical characteristics and associations between DHA, AA and serum platelet count, by PMA

Clinical characteristics	All infants	No ROP treatment needed (n = 56)	ROP treatment needed (n = 22)	*P* value
Gestational age, mean (SD), wk	25.5 (1.4)	26.0 (1.3)	24.5 (1.0)	<.001
Birth weight, mean (SD), grams	797 (223)	852 (222)	658 (160)	<.01
Male, n (%)	43 (55)	30 (54)	13 (59)	ns
Necrotising enterocolitis, n (%)	5 (6)	1 (2)	4 (18)	.008
Sepsis^a^, n (%)	22 (28)	13 (23)	9 (41)	ns
Thrombocytopenia^b^, n (%)	38 (49)	30 (36)	18 (82)	<.001
DHA, 28–40 wk*	0.23–0.4	0.00–0.26	0.54–0.68	
AA, 28–40 wkk*	0.16–0.34	0.10–0.22	0.07–0.48	
DHA, 32–36 wkk*	0.34–0.43	0.17–0.26	0.59–0.66	
AA, 32–36 wkk*	0.24–0.27	0.18–0.21	0.07–0.25	

Abbreviations: AA, arachidonic acid; DHA, docosahexaenoic acid; PMA, postmenstrual age; ROP, retinopathy of prematurity; SD, standard deviation.

a Sepsis defined as clinical symptoms and confirmed by positive blood culture, If culture indicated coagulase-negative staphylococci, diphtheroids or mixed bacterial flora, then either an elevated C-reactive protein (>20 mg/L) or an elevated Interleukin-6 (>1000 ng/L) was required.

b Thrombocytopenia defined as platelet count <100 × 10^9^/L.

* Pearson correlation coefficient ranges for selected PMA weeks.
