# Supplementary material for: Association between low fatty acid levels and platelet count in infants with Retinopathy of Prematurity
Source: Acta Paediatr. Author manuscript; Available in PMC 2021 Dec 2. (PMC8637217; doi:10.1111/apa.15406)
Supplement: 1 [file NIHMS1756982-supplement-1.pdf]

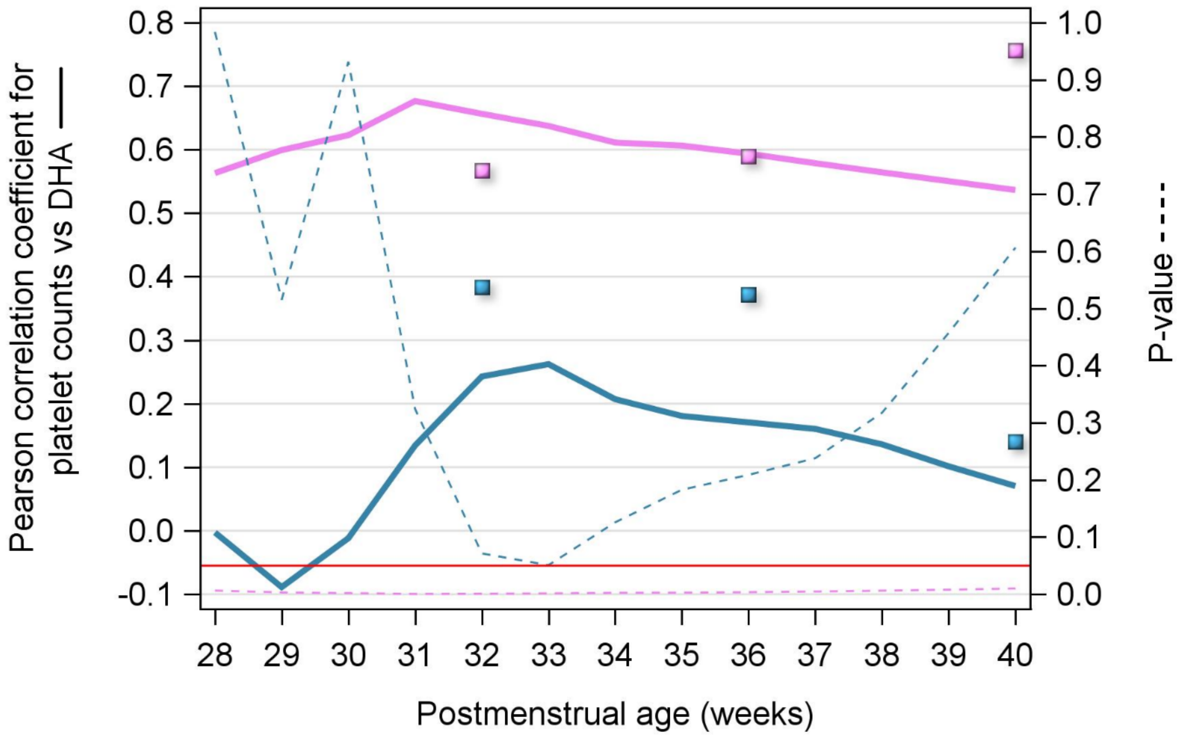

Infants with ROP treatment needed (pink lines) or not (blue lines). Colored boxes represent analyses of original values.
